# A study of the forces generated during equine cheek tooth extraction

**DOI:** 10.1002/evj.70180

**Published:** 2026-06-10

**Authors:** Tom N. A. McGinley, Richard J. M. Reardon

**Affiliations:** ^1^ The Liphook Equine Hospital Hampshire UK; ^2^ The Royal (Dick) School of Veterinary Studies The University of Edinburgh, Easter Bush Campus Midlothian UK

**Keywords:** alveolus, coronectomy, elevation, forceps, fulcrum, horse

## Abstract

**Background:**

Significant force is often applied when elevating equine teeth from the alveolus during extraction. No previous studies have investigated these forces.

**Objectives:**

To develop a method to allow measurement of elevation forces (EF) applied during extraction, and to record forces during extraction of cheek teeth in both cadaver specimens and clinical cases, evaluating factors that might affect this and comparing differences between tooth positions and horse signalments.

**Study Design:**

In vitro experimental study of cadavers and observational study of a convenience sample of clinical cases.

**Methods:**

Strain gauges were installed on extraction forceps and calibrated to give a value of force in Newtons. Cheek teeth were extracted from cadaver specimens. Two groups allowed comparison between tooth oscillation time (5 min vs. 10 min) and partial coronectomy versus no coronectomy. Measurements were also made during clinical cases presenting for cheek tooth extraction.

**Results:**

Oscillation group: Elevation forces (EF) ranged from 406.7 to 4640.4 N. The median EF was not significantly (*p* = 0.8, *V* = 187) higher in the 5‐min oscillation time group (1369.75) than the 10‐min oscillation time group (1158.95). Coronectomy group: EF ranged from 332.5 to 4015 N. The median extraction forces were significantly (*p* = 0.03) lower in the teeth that developed root fractures (947.6 N) than in the teeth that did not (1584.4 N). Clinical cases group: Elevation forces ranged between 564.3 and 2851.8 N and were significantly higher (*p* = 0.02, *W* = 114) in Maxillary (median = 1341 N) than Mandibular (median = 870 N) teeth extractions.

**Main Limitations:**

Low numbers reduced statistical power. Postmortem changes presented difficulties for ex vivo extractions, reducing validity. Conversion of input force to elevatory force and variations in fulcrum position potentially affected the accuracy of values recorded.

**Conclusions:**

High elevation forces (up to 4640.4 N in cadavers and 2851.8 N in live horses) were recorded. Elevation forces were observed to vary with tooth position, horse age, and with preparation technique.

## 1 INTRODUCTION

Cheek teeth extractions are performed commonly in equids with several indications, including endodontic, periapical and periodontal disease as well as dental fracture. Oral extraction, by which the clinical crown is grasped and manipulated by forceps within the mouth, is used most frequently and has been shown to have the lowest complication rate of cheek tooth extraction methods^1^, ^2^, ^3^; although complications still occur. Subjectively, significant force is often exerted by the operator at the final phase of oral extraction, when the tooth, grasped by forceps, is elevated from the alveolus by levering it over a fulcrum.

Complications that may be experienced during or following tooth extraction with forceps include damage to the tooth, making extraction more challenging; damage to neighbouring structures most notably neighbouring teeth, the maxilla or mandible^4^, ^5^; delayed healing due to alveolar sequestra or dry socket (non‐healing alveolus with exposed alveolar bone) formation^2^, ^4^, ^6^, ^7^ and fistula formation which can lead to further complications such as sinusitis.^2^, ^7^, ^8^

Studies have reported high masticatory forces in equids: a mean masticatory force of 875 ± 278 N was recorded in the rostral cheek teeth by one group^9^ with higher forces calculated for the caudal teeth (1613–1959 N) by another group of researchers.^10^ A recently published study measured the forces exerted on equine incisors by open mouth specula during cheek tooth odontoplasty,^11^ with median loads ranging from 198.88 to 293.95 N and a peak force of 3783.6 N being recorded. No previous studies have reported the forces required for cheek tooth extraction in equids however.

Human studies measuring extraction forces and forces leading to tooth fracture during the extraction process found that the forces that fracture teeth are sometimes only slightly higher (3%–48%) than successful extraction forces.^12^ A minimally invasive vertical extraction system used in humans recorded average maximum forces of 104 N (range 41–629 N).^13^ Due to the differences in anatomy and dental physiology between species, these findings may be of little relevance to equids.

Knowledge of the forces required to elevate an equid cheek tooth from its alveolus and the factors associated with this may help to improve extraction techniques and reduce complications both short‐ and long‐term. The aim of this study was to evaluate the forces exerted during cheek tooth elevation in equids, and investigate some factors (such as signalment, tooth position or technique employed) that influence these forces.

## 2 OBJECTIVES

There were three parts to the study:

Objective 1: Develop a method to allow accurate measurement of the elevation forces (EF) applied to equid cheek teeth during oral extraction.

Objective 2: Record EF during oral extraction of cheek teeth in cadaver specimens and evaluate factors (including cheek tooth position, time spent disrupting the periodontal attachments, and effect of partial coronectomy^14^) that might affect the amount of EF required to achieve oral extraction.

Objective 3: Record EF from clinical cases and compare differences between tooth positions, horse signalments and clinical presentation.

We hypothesised that both increased oscillation time and the performance of a partial coronectomy would result in lower EF.

## 3 METHODS

Experimental case–control study of cadavers. Observational study of a convenience sample of clinical cases.

### 3.1 Objective 1

During tooth elevation from the alveolus, a fulcrum is placed between the forceps holding the tooth being extracted and the tooth/teeth immediately rostral to it. The forceps are then used as a lever to elevate the tooth. Measuring the strain in the forceps, and accounting for the effect of the lever (with the fulcrum placed at a known distance from the held end of the forceps), it is possible to calculate the elevation force (EF) being applied to the tooth by applying the following formula (represented in Figure 1):

$$\text{Input force}\;\left(\text{IF}\right)\times \text{distance}\;1\;\left(\text{grip to fulcrum}\right)=\text{Elevatory force}\;\left(\mathrm{EF}\right)\times \text{distance}\;2\;\left(\text{fulcrum to forceps jaws}\right).$$

**FIGURE 1:**
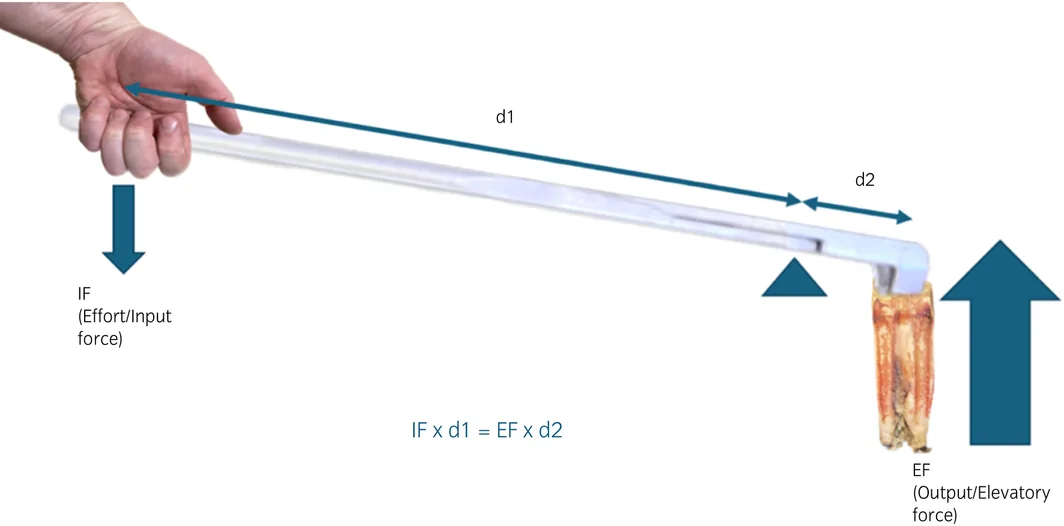
Representation of force application during cheek tooth elevation—using forceps as a lever over a fulcrum (blue arrowhead); IF = input force, d1 = distance 1, d2 = distance 2, EF = elevatory force.

Following consultation, an engineering company (Hottinger Bruel & Kjaer UK Ltd.) installed strain gauges (DY41‐3/350) on the dental extraction forceps most used by the authors (number 1 & 2 molar forceps Pegasos™) and calibrated them for input forces (as above) up to 200 N per forceps arm (total 400 N) (Figure 2). Details of how the strain gauges work and how they were calibrated are shown in Data S1. These gauges allowed measurement of strain in the forceps during tooth elevation, which was subsequently converted to give a measurement of the input force in Newtons exerted by the operator (IF).

**FIGURE 2:**
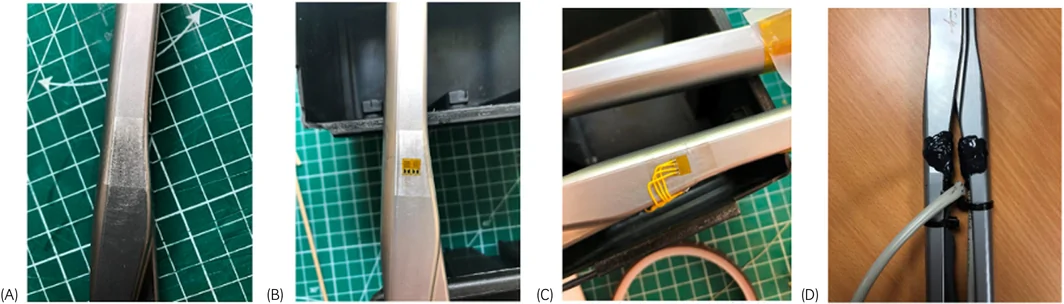
Forceps during and after installation of strain gauges: (A) preparation of forceps surface, (B) strain gauge applied, (C) cabling connected, and (D) protective resin applied.

### 3.2 Objective 2

Specimens (12 equine heads) were acquired from an abattoir and were transferred to a freezer within 3 h of acquisition and defrosted for 48–72 h before experiments. Radiographs (dorso 30° lateral—ventrolateral oblique (maxillary) and ventro 45° lateral—dorsolateral oblique (mandibular)) were taken of each cheek dental arcade for each head. The heads were classified as young (<10 years) or old (≥10 years old) based on the clinical appearance of the incisors and clinical and radiographic appearance of the cheek teeth. The heads were then randomly divided into 2 groups, with 8 in Group A undergoing comparison of forceps oscillation time and 4 in Group B undergoing evaluation of the effect of partial coronectomy.

For all heads: The jaws were disarticulated at the temporomandibular joints and the separate parts (mandible or maxilla) were subsequently placed in a vice (Yost 918‐AS) bolted to a table for extraction attempts (Figure 3).

**FIGURE 3:**
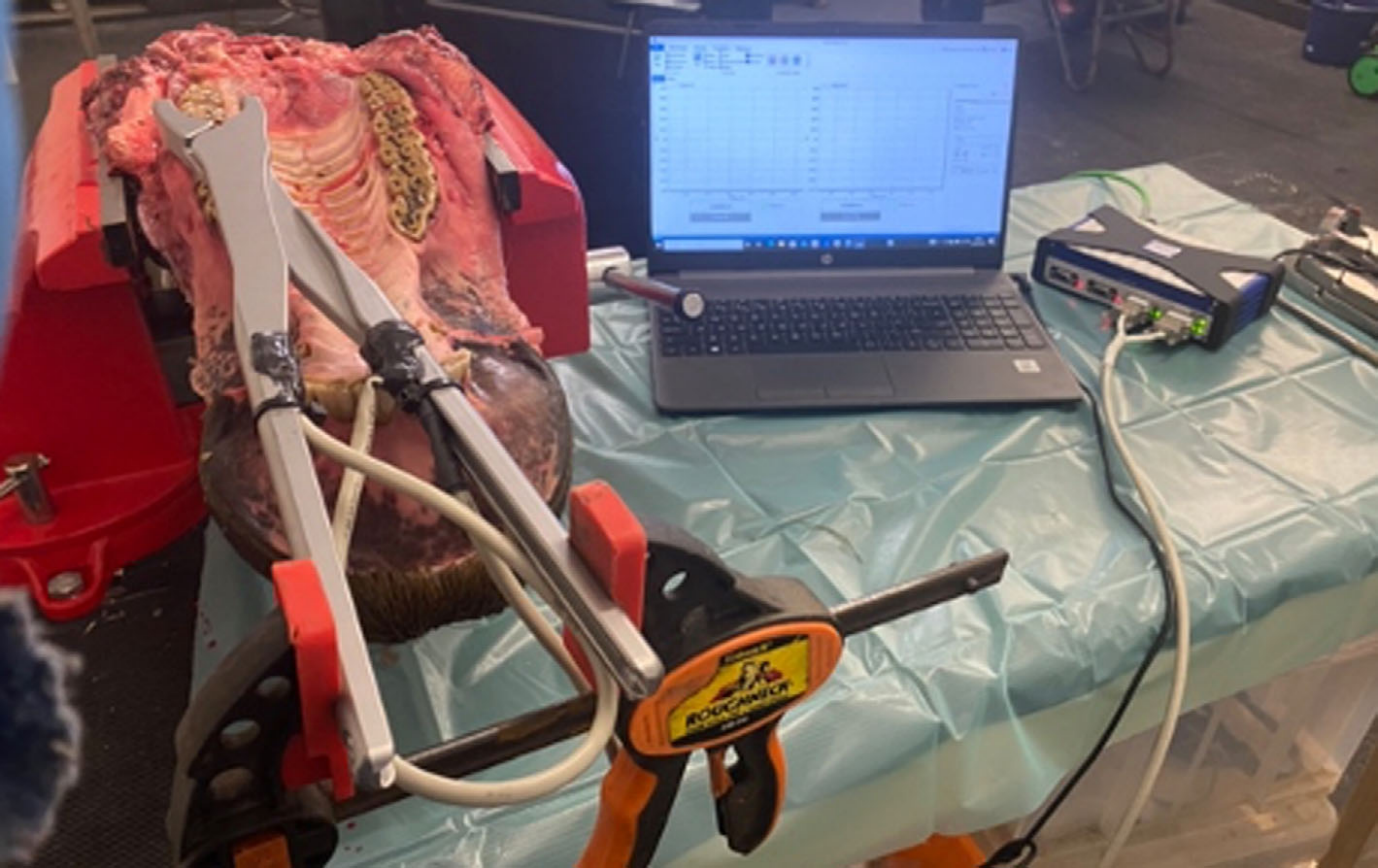
Cadaver extraction experimental setup.

Attempts were made to perform oral extraction of 2 teeth in each arcade (Triadan 09 and then Triadan 07) by the same clinician (an equine vet with >20 years' experience who currently extracts more than 100 equid cheek teeth a year) using the following sequential steps:

1. The gingiva was elevated from the periphery of the tooth using right angled elevators (Gingival elevator, Pferd dental).
2. Cheek teeth spreaders were placed either side of the tooth to be extracted with the blades of the spreaders as far apically as possible (molar separators 1 (4 mm width blades), 2 (5 mm), and 3 (6 mm) Pegasos™). Each spreader was closed and then left in place for 30 s, sequentially increasing the width of the instrument used. Spreading was performed for both the 09 s and the 07 s consecutively, and prior to oscillation of either tooth.
3. The tooth was grasped with the extraction forceps from the occlusal aspect, as far apically as possible, then the forceps were held in place using a bar clamp (ROU38256 150 mm Roughneck™) applied to the handles.
4. The forceps were moved in a lateral plane (medial and laterally), such that the tooth rotated on its long axis and held in each position (medial or lateral extent) for 0.5 s, then moved in the other direction (oscillated).
5. A fulcrum (Fulcrum set—Pferd dental) was placed 4 cm mesial to the centre of the serrated grasping jaws of the forceps, between the forceps and the teeth mesial to the one being extracted.
6. Apically directed force was then applied to the handle of the forceps, during which time strain was continuously recorded via a signal amplifier (QuantumX data acquisition unit, HBM) and recorded using data acquisition software (Catman Easy) (Figure 4) until the tooth was elevated or extraction failed because of crown fracture or adjacent tooth depression.
7. Extraction was defined as having been achieved when the tooth was extracted from the alveolus either intact or with only root fracture/fragmentation, that is, complete extraction of the clinical and reserve crown took place. All root fractures which occurred were recorded
8. Maximum force in Newtons, time to elevation (ET) in seconds, and any complications (such as tooth fracture) were recorded for each attempted extraction.
9. Maximum elevation Force (MAXEF) was calculated from the maximum force recorded by multiplying by 10.5. (Distance from handle to fulcrum/distance from fulcrum to midpoint of serrated grasping jaws).
10. Impulse (EIMP) (the mean EF multiplied by the time taken to elevate the tooth) was also calculated and recorded in Newton‐seconds.

**FIGURE 4:**
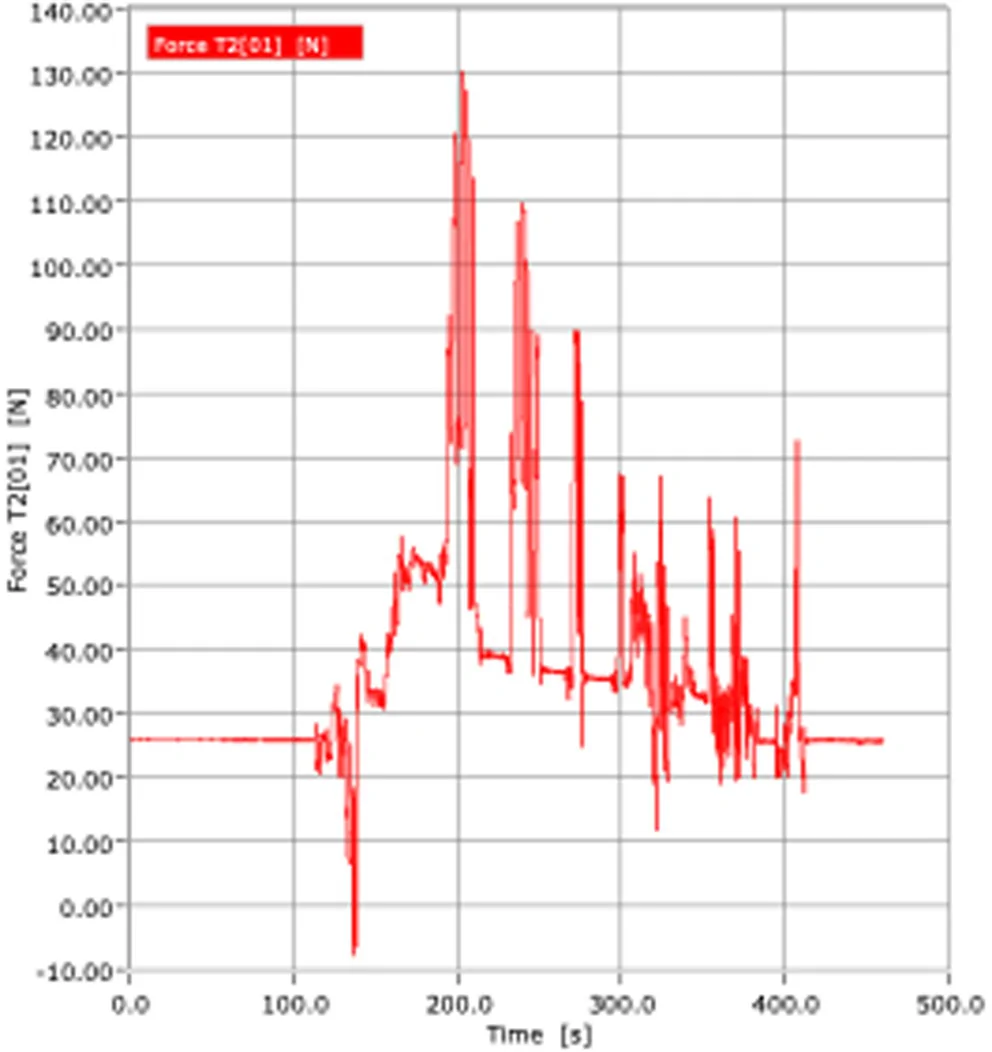
Force‐time graph for extraction of an apically infected cheek tooth (209) from a 17‐year‐old warmblood. Note the decreasing levels of force as the tooth is elevated following the initial peak as the tooth is ‘freed’ within the alveolus.

### 3.3 Group A—Effect of time of lateral oscillation prior to attempted elevation

Paired maxillary and mandibular teeth from heads in group A were randomly assigned to undergo either 5 or 10 min of oscillation (as described in number 4 above) prior to attempted elevation. For cases elevated successfully, EF and ET were compared between tooth positions (for all teeth) using Kruskal Wallis tests and between the 2 oscillation times accounting for matching using Wilcoxon signed rank tests; these comparisons were made for all teeth and between tooth positions (maxillary 07, 09, mandibular 07 and 09). Elevation forces were compared between teeth with or without root fracture using an unpaired two‐sample Wilcoxon test.

### 3.4 Group B—Effect of partial coronectomy prior to attempted elevation

Paired maxillary and mandibular teeth from heads in group B were randomly assigned to undergo partial coronectomy or not. Each extraction was performed as described points 1–4 above, having undergone 5 min of lateral oscillation (as per point 4). At that point, cases in the partial coronectomy group underwent partial coronectomy on the distal side as described by Rice and Henry^14^ using a water‐cooled IC‐300 90° angled handpiece (Equodent HDE) with a 3.175 mm carbide bur. Cheek tooth spreaders (molar separators 3 Pegasos™) were then closed across the mesial interproximal space for 30 s prior to placement of a flat right‐angled elevator (Pferd dental) in the mesial interproximal space, with force applied to distract the tooth being extracted, in a distal direction for 2 min. The rest of the steps (5–7) then proceeded as described. Representative images of the partial coronectomy technique can be seen in Figures [Link], [Link].

EF and ET were compared between extractions in the coronectomy and no coronectomy groups accounting for matching using Wilcoxon signed rank tests. Extraction forces were compared between teeth with or without root fractures using an unpaired two‐sample Wilcoxon test.

### 3.5 Part 3

Horses requiring cheek tooth extraction for clinical reasons were recruited to the study from The Liphook Equine Hospital with owner consent. Force was measured during elevation of the teeth (Figure 4), as well as clinical outcome, such as tooth fracture or other complications. Case details were recorded, including reasons for extraction and animal signalments. Extraction forces and times were compared between maxillary and mandibular tooth extractions using an unpaired two‐sample Wilcoxon test. Data analyses were performed in RStudio™.

## 4 RESULTS

### 4.1 Part 1

Application of strain gauges to the extraction forceps allowed measurement of strain with an accuracy of ±3%. Further details of the strain test results are shown in Data S1. An image of the forceps calibration rig can be seen in Figure S1.

### 4.2 Part 2

#### 4.2.1 Group A—Comparison of oscillation times

Of the 8 heads, 4 were classified as young and 4 old. Extraction was achieved in 58/64 (91%) teeth, 4 of the 6 unsuccessful extractions were due to dorsal depression of maxillary Triadan 06 teeth towards the nasal cavity during attempted elevation of maxillary 07 teeth, the other 2 failed due to reserve crown fracture (Triadan 407 tooth) and clinical crown fracture (Triadan 309 tooth). Roots and/or root tip fractures occurred in 23 teeth (12 in the 5 min and 11 in the 10‐min groups; 9 maxillary/14 mandibular), in one mandibular tooth extraction (Triadan 407 in an ‘old’ horse) both roots fractured during manipulation which allowed extraction without the use of a fulcrum, so no elevation strain was recorded. These data allowed 26 contralateral paired tooth (13 mandibular and 13 maxillary) MAXEF comparisons, of these, time was not recorded for 6 cases, allowing ET and EIMP comparisons for 20 contralateral paired teeth (10 mandibular and 10 maxillary).

MAXEF across all extracted teeth ranged from 406.7 to 4640.4 N. There were non‐significant (*p* = 0.08) variations in MAXEF between extracted tooth positions, irrespective of oscillation group (Figure 5). The median MAXEF was not significantly (*p* = 0.8, *V* = 187) higher in the 5‐min oscillation time group (1369.75) than the 10‐min oscillation time group (1158.95) and MAXEF did not differ significantly between oscillation time groups for any of the tooth positions (Table 1 and Figure 6). The median MAXEF was not significantly (*p* = 0.4) higher in the teeth that developed root fractures (1701 N) than in the teeth that did not (1204 N).

**FIGURE 5:**
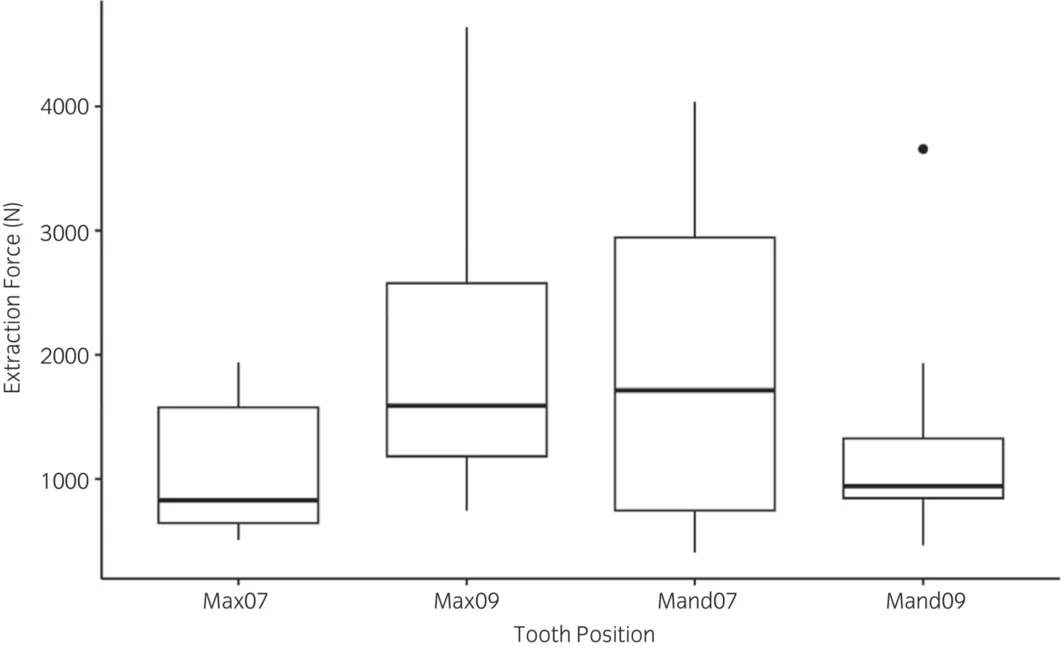
Maximum extraction forces at each tooth position for the 26 teeth compared in part 2A of the study. Lower and upper box lines = 25th and 75th percentiles, respectively; Middle box line in bold = median; lower and upper whiskers = lower and upper adjacent values, respectively; dots = outliers. Max07 = maxillary Triadan 07 teeth, Max 09 = maxillary Triadan 09 teeth, Mand07 = mandibular Triadan 07 teeth, Mand09 = mandibular Triadan 09 teeth.

**TABLE 1:** Median extraction forces in Newtons (N) and time in seconds (S) between tooth positions and oscillation times, with results of Wilcoxon signed rank (WSR) test comparisons between 5‐ and 10‐min oscillation times (*V* = test statistic).

Tooth	*N* per group	Oscillation time (min)		WSR test results 5 v 10	
		5	10	*V*	*p*‐value
Median ‘maximum elevation force’ (N)					
Maxillary 07	5	815.1	838.6	6	0.8
Maxillary 09	8	1588.3	1677.3	19	0.9
Mandibular 07	7	1806.4	1318.9	19	0.5
Mandibular 09	6	1004.9	942.1	10	>0.9
Median elevation impulse (NS)					
Maxillary 07	5	29,222.26	34,239.67	2	0.2
Maxillary 09	5	39,891.35	34,878.34	15	0.06
Mandibular 07	6	12,001.96	7022.80	15	0.4
Mandibular 09	4	11,331.26	5842.66	10	0.1
Median extraction time (S)					
Maxillary 07	5	102	95.2	6	0.8
Maxillary 09	5	124.4	128.7	10	0.6
Mandibular 07	6	96.15	97.35	6	0.4
Mandibular 09	4	113.25	131.7	2	0.4

**FIGURE 6:**
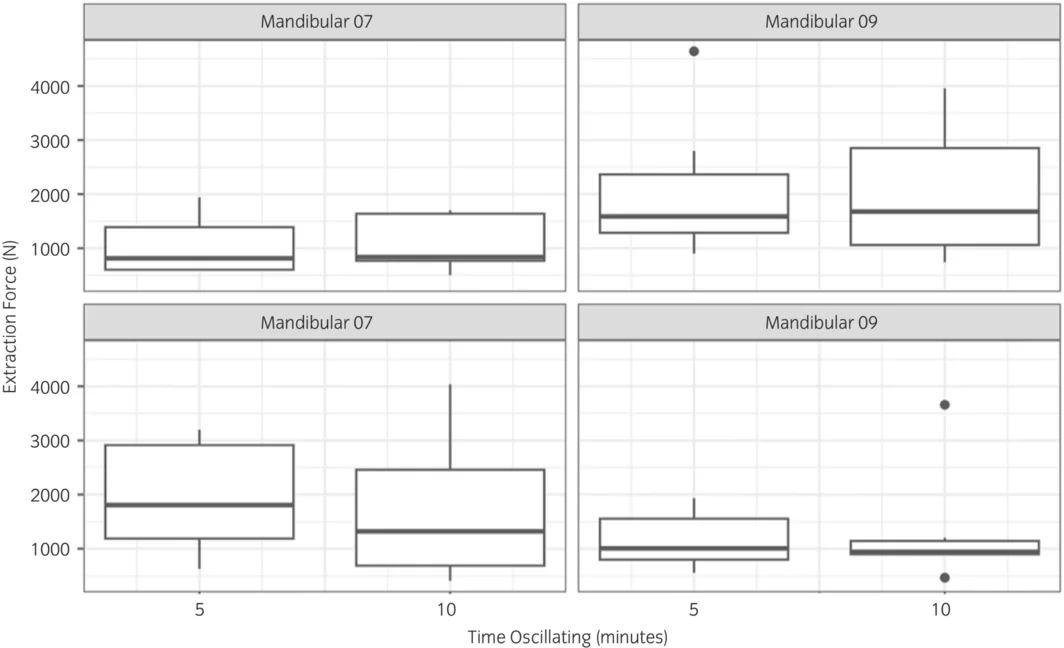
Maximum extraction forces from 26 teeth between different tooth positions and oscillation times. Lower and upper box lines = 25th and 75th percentiles, respectively; Middle box line in bold = median; lower and upper whiskers = lower and upper adjacent values, respectively; dots = outliers.

EIMP across all extracted teeth ranged from 2340 to 113,597 NS. There were significant (*p* < 0.001) variations in EIMP between extracted tooth positions (irrespective of oscillation group), with mandibular teeth having significantly lower EIMP than maxillary. The median EIMP was significantly (*p* < 0.001, *V* = 210) higher in the 10‐min oscillation time group (23,782 NS) than the 5‐min oscillation time group (21,034 NS), but EIMP did not differ significantly between oscillation time groups for any of the tooth positions (Table 1). The median EIMP was not significantly (*p* = 1) lower in the teeth that developed root fractures (15,807 NS) than in the teeth that did not (29,222 NS).

ET across all extracted teeth ranged from 75 to 365.6 s. There were significant (*p* = 0.005) variations in ET between extracted tooth positions (irrespective of oscillation group), with Maxillary Triadan 09 s having significantly longer times than Maxillary Triadan 07 s (*p* = 0.02) and Mandibular Triadan 07 s (*p* = 0.02). The median ET was not significantly (*p* = 0.6, *V* = 89) higher in the 10‐min oscillation time group (113.7S) than the 5‐min oscillation time group (105.8S) and ET did not differ significantly between oscillation time groups for any of the tooth positions (Table 1).

#### 4.2.2 Group B—Effect of partial coronectomy

Of the 4 heads, 2 were classified as young and 2 old. Extraction was achieved for all (32) teeth. Roots and/or root tip fractures occurred in 14 teeth (6 in the coronectomy and 8 in the non‐coronectomy groups; 7 maxillary/7 mandibular); in one mandibular tooth extraction (Triadan 107 in an ‘old’ horse), the mesial and palatal roots fractured during manipulation, which allowed extraction without the use of a fulcrum, so no elevation strain was recorded. In one specimen, the 307 and 407 were depressed apically during prior extraction of the 309 and 409, so their subsequent extraction required minimal effort, and these forces were not included for comparisons. These data allowed 14 contralateral paired tooth (7 mandibular and 7 maxillary) Elevation force and Time comparisons.

MAXEF across all extracted teeth ranged from 332.5 to 4015 N. The median MAXEF was higher in the non‐coronectomy group (1400 N) than the coronectomy group (1098 N) but not significantly so (*p* = 0.2, *V* = 73) and did not differ significantly between coronectomy groups for any of the tooth positions (Table 2). The median MAXEF was significantly (*p* = 0.03) lower in the teeth that developed root fractures (947.6 N) than in the teeth that did not (1584.4 N).

**TABLE 2:** Median elevation forces and times between tooth positions and coronectomy no/yes, with results of Wilcoxon signed rank (WSR) test comparisons between coronectomy no/yes (*V* = test statistic).

Tooth	*N* per group	Coronectomy		WSR test results 5 v 10	
		No	Yes	*V*	p‐value
Maximum ‘median extraction force’ (N)					
Maxillary 07	3	1404.9	1163.0	2	0.8
Maxillary 09	4	1408.7	1425.4	3	0.6
Mandibular 07	3	1071.4	862.3	6	0.3
Mandibular 09	4	1438.9	114.75	9	0.3
Median ‘elevation impulse’ (NS)					
Maxillary 07	3	30,459.7	33,782.1	3	>0.9
Maxillary 09	4	47,280.3	65,805.6	5	>0.9
Mandibular 07	3	14,139.9	11,832.7	5	0.5
Mandibular 09	4	14,772.3	24,241.8	3	0.6
Median extraction time (S)					
Maxillary 07	3	92.8	94.6	3	>0.9
Maxillary 09	4	187.4	158.2	10	0.1
Mandibular 07	3	101.1	108.7	3	>0.9
Mandibular 09	4	124.8	154.3	3	0.6

EIMP across all extracted teeth ranged from 5130 to 128,375 NS. The median EIMP was higher in the non‐coronectomy group (35,017.8 NS) than the coronectomy group (24,680.4 NS) but not significantly so (*p* = 0.95, *V* = 54) and did not differ significantly between coronectomy groups for any of the tooth positions (Table 2). The median EIMP was not significantly (*p* = 0.08) lower in the teeth that developed root fractures (15,957.49 NS) than in the teeth that did not (38,338.1 NS).

ET across all extracted teeth ranged from 49.5 to 513.7 s. The median ET was higher in the non‐coronectomy group (122.7 S) than the coronectomy group (104.0 S) but not significantly so (*p* = 0.6 *V* = 62) and did not differ significantly between coronectomy groups for any of the tooth positions (Table 2).

### 4.3 Part 3

Extraction forces were measured for 31 successful oral cheek tooth extractions from 24 live horses (20 maxillary and 11 mandibular teeth). Apical infection was the most common diagnosis in 61.3% (19/31) of cases, with periodontal disease in 35.4% (11/31) and dental fractures in 25.8% (8/31) of teeth; horse ages ranged from 7 to –23 years (median 13 years) and a variety of breeds were treated, details of which are shown in Table S1. Root fractures occurred in 5 cases (3 mandibular, 2 maxillary), with the root fragments subsequently retrieved from the alveoli in all, either digitally or using right angled elevators.

MAXEF recorded from all 31 extractions ranged between 564.3 and 2851.8 Newtons and was significantly higher (*p* = 0.02, *W* = 114) in Maxillary (median = 1341 N) than Mandibular (median = 870 N) teeth extractions – shown graphically in Figure S4. MAXEF varied by tooth positions (Figure 7) and with horse ages (Figure S5).

**FIGURE 7:**
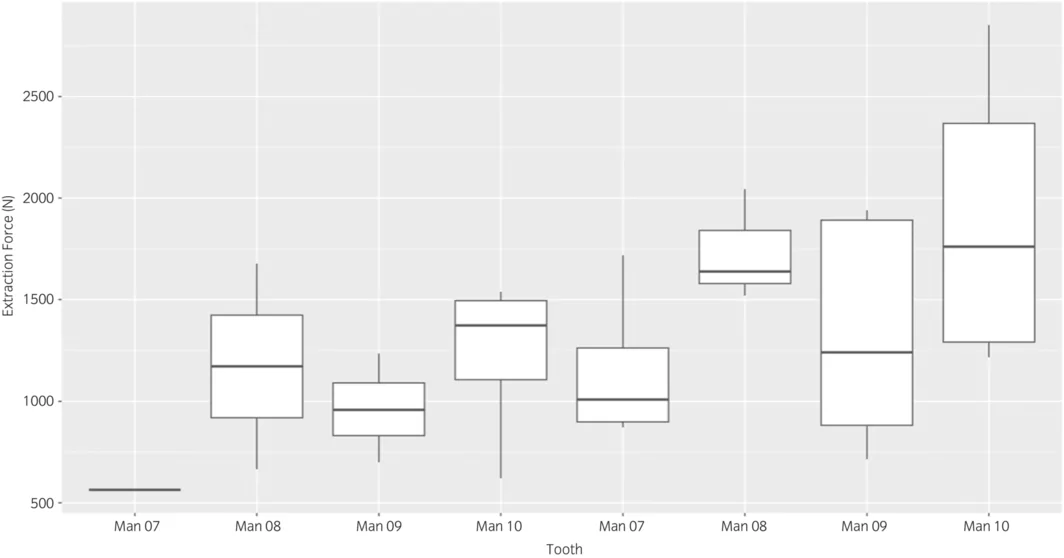
Maximum elevation forces recorded at each tooth position from 31 clinical cases. X‐axis: Man = mandibular, Max = maxillary, numbers = Triadan positions. Lower and upper box lines = 25th and 75th percentiles, respectively; Middle box line in bold = median; lower and upper whiskers = lower and upper adjacent values, respectively; dots = outliers.

EIMP could be calculated for 22 cases and ranged between 24,298 and 1,287,385 NS and was significantly higher (*p* = 0.05, *W* = 91) in Maxillary (median = 124,479.6 NS) than Mandibular (median = 52,859.5 NS) teeth extractions – shown graphically in Figure S6. Elevation Impulse varied by tooth positions and with horse ages (Figure S7).

ET from 22 live cases ranged between 181.5 and 3183.8 Seconds (~3 to 53 min) and were not significantly higher (*p* = 0.3, *W* = 40) in Mandibular (median = 496.3 S) than Maxillary (median = 365.0 S) teeth extractions – shown graphically in Figure S8. ET varied by tooth positions and with horse ages (Figure S9).

## 5 DISCUSSION

To our knowledge, this is the first published study reporting cheek tooth elevation forces in equids. Elevation force was measured successfully in both cadavers and live equids by using strain gauges mounted on the extraction forceps and measuring the distance from the fulcrum (lever length). Other techniques to measure elevation force were trialled, including use of a pressure sensor in the place of a fulcrum, but during a pilot study, positioning the fulcrum proved unreliable due to instability.^15^ Use of strain gauges mounted on the forceps conferred several advantages: Firstly, due to the sensitivity of the gauges, it was possible to make highly accurate readings (estimated ±3%—see Data S1 for further detail). Secondly, as two gauges were mounted on each arm of the forceps, and the sum for each arm gave the total force required to extract the tooth, we were able to make a measurement of purely vertical force, with any rotation in the forceps being negated. The third, significant advantage of using strain gauges is that they are extremely small and discrete, meaning that extractions could be performed using the forceps as normal in clinical cases.

Calculation of EF was reliant on knowing the fulcrum position in relation to the forceps. This was easier to control in cadavers, but may have varied slightly in clinical cases due to the dynamic nature of the procedure and more limited access (preventing accurate measurement and so affecting the results). For example, a change in distance of the fulcrum from the forceps jaws by 0.5 cm mesially or distally would have resulted in a 13% reduction or 14.5% increase in EF respectively, such is the amplificatory effect of a lever. Additionally, using our calculation we have assumed 100% transfer of force, and since some apically directed force is also likely to be transferred through the fulcrum itself, true values applied to the teeth can be assumed to be slightly lower. The values provide a useful estimation of representative forces; however.

A wide range of MAXEF was recorded, with variations between tooth positions, extraction techniques, horse signalments and clinical presentation (Table S1). Maximum recorded elevation forces were reported in this study, as these were considered a simple way of describing how much force was used. Cheek tooth extraction can often require prolonged force application however. As such, Impulse (a measurement of force over time) was also recorded, to provide additional information about these cumulative forces, although unfortunately did not record successfully for all cases.

Cadaver heads were used for part 2 of the study, to confirm efficacy of the strain gauges prior to use in clinical cases and to facilitate comparisons between techniques using matching controls. There are several potential limitations of cadaver use, the most pertinent being potential alterations in the tissues (particularly the periodontal tissues) between cadavers and live animals. The experimental setup cannot accurately replicate a real‐life extraction process, with specimens disarticulated and mounted in a vice. This will have led to variations in the angles and forces applied. Subjectively, effort was frequently more than that which one would feel comfortable exerting in a live case, due to the need to attempt extraction at standardised timings, regardless of the mobility achieved in preparation. Teeth were oscillated laterally (~2hz) by one operator, at a level of pressure designed to replicate that which might occur in a clinical setting and to be comparable in each case. However, in clinical cases, the level of pressure applied, as well as the rate of oscillation will vary throughout, dependant on the presenting findings and the palpable feedback intra‐procedure. The periodontal ligament is also often further disrupted by holding the tooth at the end of range for a sustained period, and many operators use this as an additional method for disrupting the periodontal ligament, although this was not done in our cadaver experiments. In vivo, the forceps may also be removed and further elevation, spreading or odontoplasty performed to aid extraction. Another departure from the in vivo situation was that two teeth were extracted from each arcade, in order to maximise the data obtained from the specimens which were available. This may have affected the forces required to elevate the 07 cheek teeth in particular, since there would have been more effective 07–08 spreading once the 09 was no longer present and providing a splinting effect. In order to minimise this potential effect, spreading was performed in all four interproximal spaces (06–07, 07–08, 08–09, 09–10) prior to any attempted extraction (meaning that the 09 remained present during 07–08 spreading).

In the cadaver experiments, 3 sets of spreaders were used and fully closed in every case (molar separators 1, 2 & 3 Pegasos™) in an effort to standardise the procedure as much as possible. In clinical cases, however, use of specific spreaders and indeed how they are used will vary, dependent on case specifics and tactile feedback during the procedure. The third set of spreaders (blade width 6 mm) distract teeth by the largest distance, and it was considered that consistent application of this instrument in the cadaver experiments may have led to an increased incidence of root fracture.

MAXEF recorded in the cadaver specimens ranged from: 332.5 N (in a 309, that had diastemata either side, extraction in the partial coronectomy comparisons) to 4640.4 N (in a 109, that had root dilaceration, extraction in the oscillation comparisons) and spanned those recorded in the live cases: 564.3 N (in a 307 extraction in a 9 year old Thoroughbred mare) to 2851.8 N (in a 110 extraction in a 7‐year‐old Irish Draught gelding). The highest MAXEF recorded (4640.4 N) was equivalent to an input force of 441.9 N, which is 10.5% higher than the force for which the strain gauges were calibrated (400 N). This was one of only three cases out with this range, and following further consultation with HBK engineers, it was not thought likely to have significantly affected the accuracy of these recordings. The large range of extraction forces recorded across the study makes it hard to ascertain the effect of postmortem tissue changes, although potentially the absolute forces recorded from cadavers are less relevant to the clinical situation.

ET recorded in the cadaver specimens ranged from 50 to 514 S and from 182 to 3184 S (~53 min) in live horses. The longer elevation times in clinical cases are likely to reflect the additional care taken, and the challenging nature of extraction in certain cases.

EIMP recorded in the cadaver specimens ranged from 5130 to 128,375 NS in cadavers and 24,298 to 1287,385 NS in live horses. The greater EIMP values reflect the longer elevation times in clinical cases. It is not known whether these large cumulative forces represent any further risk to the patient, given the robust nature of the periodontal tissues and their adaptation to withstand the large and repetitive forces of mastication.^9^, ^10^

EIMP in clinical cases was significantly higher (*p* = 0.05, *W* = 91) in Maxillary (median = 124,479.6 NS) than Mandibular (median = 52,859.5 NS) teeth extractions. This may reflect differences in anatomy such as tooth shape and root morphology or pre‐existing disorders such as periodontal disease, which were much more prevalent for the mandibular teeth.

The highest recorded MAXEF, ET and EIMP in a live patient were all recorded for the same extraction which was subjectively observed to be challenging (a rotated 110 extraction in a 7‐year‐old Irish Draught gelding). These values are likely to reflect the difficulty of this specific case.

In four cadaver specimens (all classified as young, i.e., <10 years old) bilaterally in two cases, apical depression of the tooth on which the fulcrum was resting occurred during attempted elevation: during maxillary 07 elevation in 3 cases: 106 (1999 N) and 206 (2482 N) (*n* = 1), 106 (1693.4) (*n* = 1), 206 (4111 N) (*n* = 1) and during attempted mandibular 09 elevations both 07 s (4015 N and 2848 N) were depressed apically (*n* = 1). The forces recorded leading to apical depression of neighbouring teeth were towards the top end of the figures recorded from live cases. Tooth depression is an uncommon but reported sequelae of cheek tooth extraction.^7^ The frequency (4/12) observed in the cadaver heads subjectively would not be representative of the situation in live cases, and may be related to postmortem changes to the tissues such as compromised integrity of the periodontal ligament and alveolar bone. This potential complication should be considered however when applying elevatory force to teeth during extractions, in particular rostral maxillary teeth in young animals. Consideration might be given to the use of a reserve fulcrum (a short extension attached to the grasping end of the forceps, under which a narrow standard fulcrum can be placed, allowing leverage forces to be applied distal rather than mesial to the tooth being extracted) in such cases, to reduce the pressure exerted on one tooth.

Use of matched controls (comparing teeth from contra‐lateral arcades) within the same specimens in Part 2 was designed to minimise potential confounding factors, such as variation between breeds, ages, teeth positions and postmortem tissue changes. However, some variations between tested teeth, such as degree of periodontal disease, may have affected the results. Variations such as these make comparisons of extraction forces between the live cases more difficult to interpret. For example, the significantly lower EF recorded for mandibular than maxillary teeth extractions in the clinical cases may have been influenced by the 91% (10/11) of mandibular extraction cases that had periodontal disease compared to only 5% (1/20) of the maxillary extractions.

Longer oscillation periods were expected to disrupt the periodontal attachments more effectively and result in lower elevation forces. Although there was a trend for lower elevation forces in the 10 than the 5‐min oscillation group, this difference was not significant. This lack of significance may be related to the sample size but could also be related to the relatively short oscillation times investigated. These oscillation times were chosen as considered to be most relatable to the clinical situation.^16^ Future studies comparing longer oscillation times may provide useful information in this area.

Use of partial coronectomy has been reported to improve oral extractions in equids, subjectively reducing the elevation forces applied to the clinical crown.^14^, ^17^ In this study, there was a trend, but not significant, with median elevation forces being lower in the teeth that had undergone coronectomy than those that had not. The lack of statistical significance may be related to the small sample size investigated and may benefit from further investigation.

Root fractures occurred only in teeth with root formation at their apex (i.e., not in very young teeth). There were higher frequencies of root fractures in the cadaver tooth extractions: 36% (23/64) in the oscillation comparisons and 44% (14/32) in the coronectomy comparisons, in contrast to 16% (5/31) of clinical case extractions. The reasons for this may be related to alterations in cadaveric tissues compared to vital ones, differences caused by disease in the clinical cases or differences in technique such as prolonged tooth preparation and oscillation, and more careful/nuanced elevation in the clinical cases.

Across all groups, the incidence of root fracture was higher in mandibular: 43.9% (25/57) than maxillary: 27.7% (18/65) cheek teeth. This was also true for the live cases, with root fracture occurring in 27.2% (3/11) of mandibular cheek tooth extraction versus 10% (2/20) of maxillary cheek tooth extractions. This may reflect the longer roots present on mandibular cheek teeth, and the subjectively more challenging extraction procedures. It should be considered, however, that only teeth in which oral extraction with forceps was successful, and could be concluded using the experimental forceps, are included in these figures. In the cadaver comparisons, root fracture was associated with not‐significantly higher median extraction forces in the oscillation time comparisons group but significantly lower median extraction forces in the coronectomy comparison group. Higher extraction forces may predispose to root fracture and be necessary for cases with root dilaceration; conversely, root fracture may facilitate easier elevation, which may partly explain some of the observed differences here. Further investigations of forces in oral extractions, including forces applied prior to failed extraction attempts, extractions completed with different types of forceps, and/or investigation of alternative extraction techniques such as reverse fulcrum use, would be interesting.

In conclusion, the described experimental model is effective and could be applied to a larger number of specimens and clinical cases. High elevation forces (up to 4640.4 N MAXEF in cadavers and 2851.8 N in live horses) were recorded in this study. Prolonged force exertion was also a feature in many cases (ET up to 514 s in cadavers and 3184 s in live horses, with resultant EIMP up to 128,375 Ns in cadavers and 1,287,385 Ns in live horses). Complications associated with these large and sometimes prolonged forces, such as damage to surrounding teeth and periodontal structures, should be considered when performing oral cheek tooth extractions in equids. In live horses, MAXEF required to elevate Maxillary cheek teeth was significantly higher (median = 1341 N) than that required to elevate Mandibular cheek teeth (median = 870 N). This may reflect tooth and root morphology and may also be influenced by specific disease processes such as periodontal disease. Elevation forces were observed to vary with tooth position, horse age and with preparation techniques such as oscillation and coronectomy. Coronectomy may lead to a reduction in the EF required for extraction although further studies are needed to confirm these findings.

## FUNDING INFORMATION

The project was funded by the Horse Trust. Project ref. G2022.

## CONFLICT OF INTEREST STATEMENT

The authors declare no conflicts of interest.

## AUTHOR CONTRIBUTIONS

**Tom N. A. McGinley:** Funding acquisition; conceptualization; methodology; project administration; data curation; resources; writing – original draft; investigation; validation; visualization; writing – review and editing. **Richard J. M. Reardon:** Conceptualization; writing – review and editing; formal analysis; supervision; methodology; data curation; writing – original draft.

## DATA INTEGRITY STATEMENT

Tom N. A. McGinley had full access to all the data in the study and takes responsibility for the integrity of the data and the accuracy of data analysis.

## ETHICAL ANIMAL RESEARCH

The methodology described for data collection was approved by the RCVS Ethics Review Panel. Ref. 2023‐099‐McGinley.

## INFORMED CONSENT

Informed consent was given by the owners for horses included in the study.

## Supporting information


**Data S1.** Force measurement.

**Table S1.** Signalments, diagnoses, extracted teeth and maximum elevation forces for the 31 cases in Part 3.

**Figure S1.** Forceps calibration rig.

**Figure S2.** Maxillary 07 tooth post coronectomy (blue arrow), gross appearance.

**Figure S3.** Maxillary 07 tooth post coronectomy (blue arrow), radiographic appearance.

**Figure S4.** Maximum elevation forces recorded from 31 live cases subdivided by dental arcades.

**Figure S5.** Maximum elevation forces recorded from 31 live cases subdivided by age and dental arcades.

**Figure S6.** Impulse elevation forces recorded from 22 live cases subdivided by dental arcades.

**Figure S7.** Elevation impulse recorded from 22 live cases subdivided by age and dental arcades.

**Figure S8.** Elevation times recorded from 22 live cases subdivided by dental arcades.

**Figure S9.** Elevation times recorded from 22 live cases subdivided by age and dental arcade.

## Data Availability

The data that support the findings of this study are openly available in ‘A study of the forces generated during equine cheek tooth extraction—Dataset’ at https://figshare.com/s/5156bc71279d6ab39d36.
