# Supplementary material for: A study of the forces generated during equine cheek tooth extraction
Source: Equine Vet J. 2026 Jun 10;58(6):1579–89. doi: 10.1002/evj.70180 (PMC13657190; doi:10.1002/evj.70180)
Supplement: Supplementary file 3 [file EVJ-58-1579-s003.pdf]

Equine Veterinary Journal BEVA

Table S1: Signalments, diagnoses, extracted teeth and Maximum Elevation Forces for the 31 cases in Part 3.

Horse	Age (years)	Breed	Sex	Diagnosis	Tooth (Triadan)	Max Elevation Force (N)
1	19	Welsh D	G	CCF	210	1316.1
2	19	Fell pony	G	AI	209	1892.3
3	13	Connemara X	G	AI	210	2206.1
4	13	Connemara X	G	AI	109	714.2
5	8	ISH	M	CF & AI	208	2044.5
6	10	Cob	G	CCF & AI	209	1927.9
7	10	Cob	G	AI	109	1240.4
8	13	Welsh X	M	AI	109	1046.9
9	12	TB	G	BSF & AI	210	1216.7
10	22	Arab x	M	Lingual displacement & PD	409	698.8
11	22	Arab x	M	Buccal displacement & PD	410	621.6
12	7	Irish draught	G	Rotated tooth & PD	110	2851.8
13	22	Haflinger	M	AI	207	1110
14	16	ISP	G	BSF & AI	107	907.5
15	16	ISP	G	BSF & AI	207	871.5
16	9	TB	M	AI. Detached distal root tip	307	564.3
17	10	Welsh D	G	AI	108	1638.5
18	13	SH	G	Diastemata & severe PD	308	1677.3
19	13	SH	G	Diastemata & severe PD	408	665.9
20	12	ISH	G	AI	109	881.6
21	22	New Forest X	M	Diastemata & PD	409	1041.2

22	10	Welsh D	G	AI	107	1719.1
23	22	Cob	G	CF & AI	109	1939.9
24	17	Danish WB	G	AI	209	1366.2
25	20	Arab	G	Diastemata & PD	409	1234.7
26	20	Arab	G	Diastemata & PD	410	1267.0
27	20	Arab	G	Diastemata & PD	309	874.7
28	20	Arab	G	Diastemata & PD	310	1480.2
29	13	WB	G	AI	208	1519.9
30	23	WB	M	Diastemata & PD	310	1538.9
31	13	WB	M	CF & AI	209	873.5

Key: X=cross, ISH=Irish Sports Horse, TB=Thoroughbred, ISP=Irish Sports Pony, SH=Sports Horse, WB=Warmblood, G=Gelding, M=Mare, CCF=Complicated Crown Fracture, AI=Apical Infection, CF=Crown Fracture, BSF=Buccal Slab Fracture, PD=Periodontal Disease.
