# Supplementary material for: A study of the forces generated during equine cheek tooth extraction
Source: Equine Vet J. 2026 Jun 10;58(6):1579–89. doi: 10.1002/evj.70180 (PMC13657190; doi:10.1002/evj.70180)

Equine Veterinary Journal
BEVA

Figure S5: Maximum Elevation Forces recorded from 31 live cases subdivided by age and dental arcades.

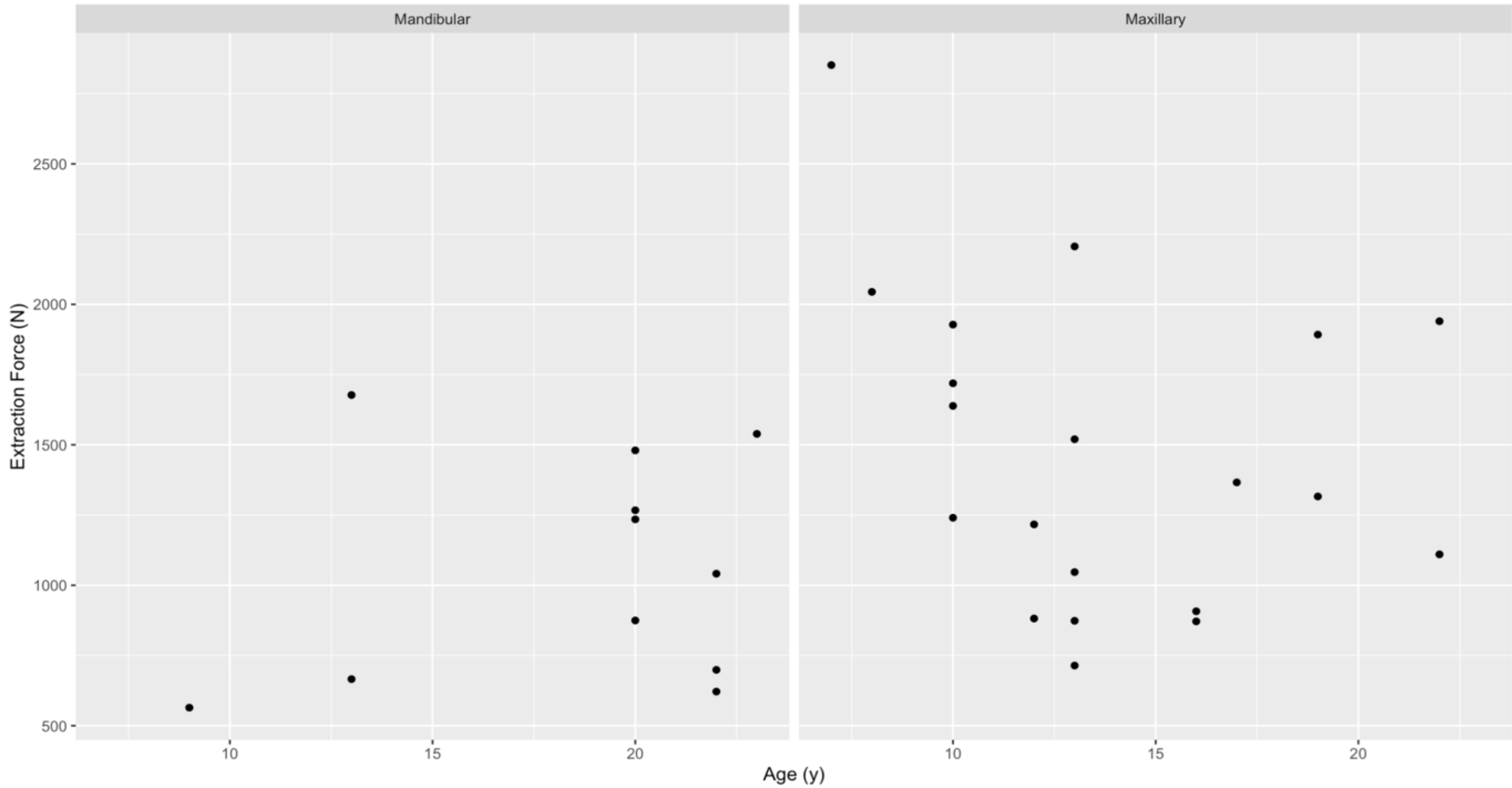

Supplement: Supplementary file 5 [file EVJ-58-1579-s005.pdf]
