# Supplementary material for: A study of the forces generated during equine cheek tooth extraction
Source: Equine Vet J. 2026 Jun 10;58(6):1579–89. doi: 10.1002/evj.70180 (PMC13657190; doi:10.1002/evj.70180)

Equine Veterinary Journal BEVA

Figure S4: Maximum Elevation forces recorded from 31 live cases subdivided by dental arcades. Lower and upper box lines=25th and 75th percentiles, respectively; Middle box line in bold=median; lower and upper whiskers=lower and upper adjacent values, respectively; dots=outliers.

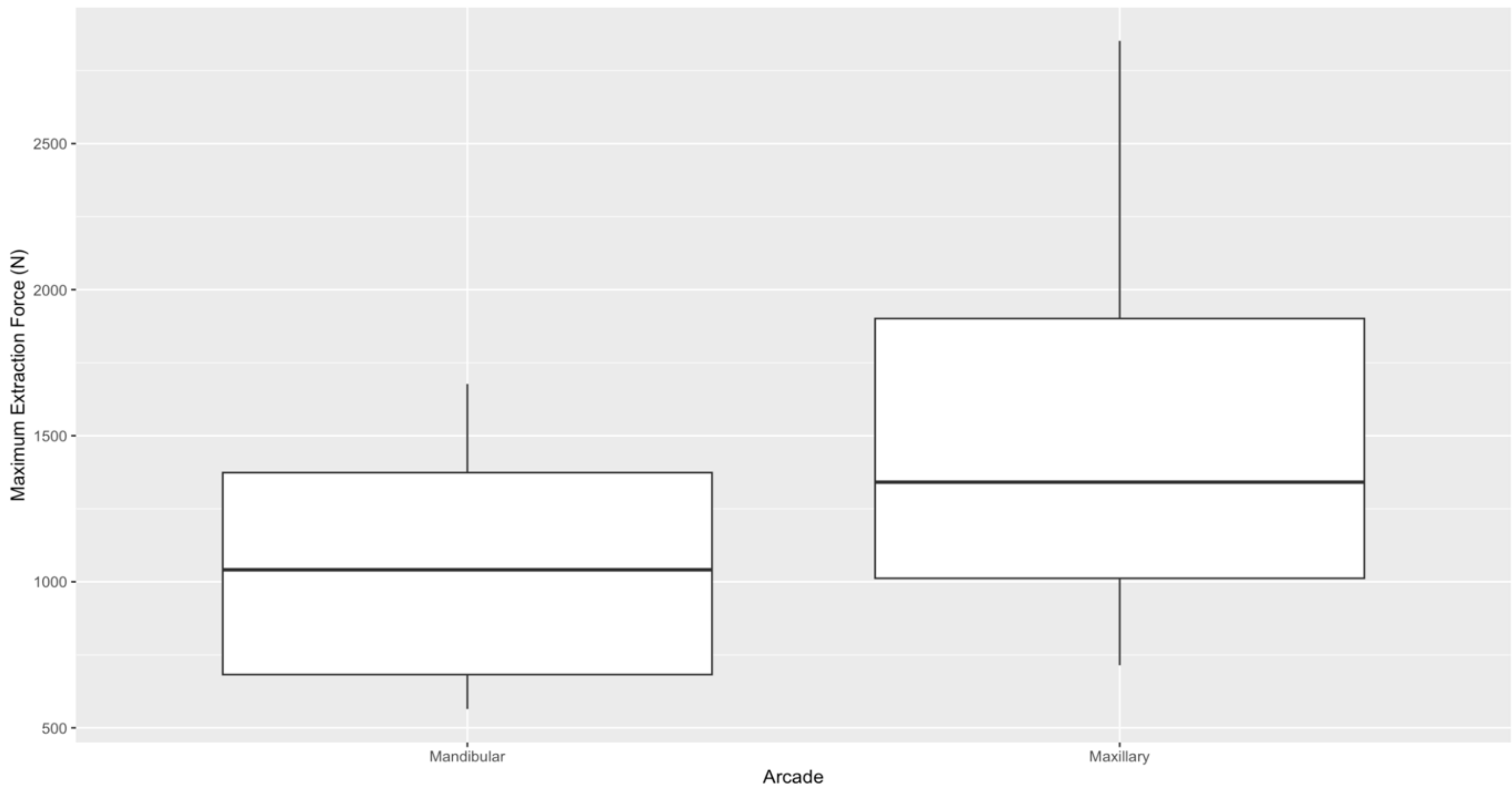

Supplement: Supplementary file 6 [file EVJ-58-1579-s006.pdf]
