# Supplementary material for: A study of the forces generated during equine cheek tooth extraction
Source: Equine Vet J. 2026 Jun 10;58(6):1579–89. doi: 10.1002/evj.70180 (PMC13657190; doi:10.1002/evj.70180)

Equine Veterinary Journal BEVA

Figure S3: Maxillary 07 tooth post coronectomy (blue arrow), radiographic appearance.

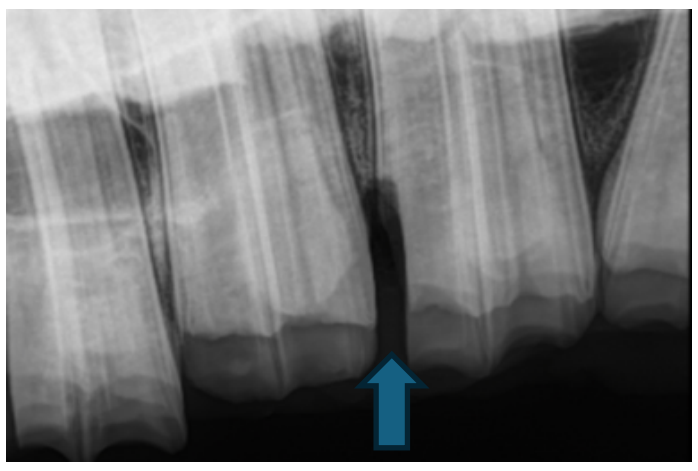

Supplement: Supplementary file 8 [file EVJ-58-1579-s008.pdf]
