# Supplementary material for: A study of the forces generated during equine cheek tooth extraction
Source: Equine Vet J. 2026 Jun 10;58(6):1579–89. doi: 10.1002/evj.70180 (PMC13657190; doi:10.1002/evj.70180)
Supplement: Supplementary file 9 [file EVJ-58-1579-s009.pdf]

Equine Veterinary Journal BEVA

Data S1: Force measurement

How strain gauges work:
Strain gauges are small sensors. The strain gauge is adhered to a surface, and as the surface elongates and compresses, so does the strain gauge. This causes the resistance value in the strain gauge to change. By connecting the strain gauge in a Wheatstone bridge; the change in resistance is measured in mV/V. The mV/V signal is then converted to strain [μm/m]. The forceps were gauged in a bending configuration.

Strain gauge calibration:
A load cell was used to correlate strain measurement to a force measurement for each arm of the extraction forceps. Each arm was loaded with up to 200N of force to create bending in the arm as would occur during an extraction. The data collected allowed each arm to be calibrated and a calibration chart produced for each individual arm. The sum of each arm gave the total force required to extract the tooth.

Sensor assessment
Several tests were made to assess the integrity of the strain gauge installation.

Resistance check:
A resistance measurement is done before covering each strain gauge. The measurement was done with a multimeter.
• Strain gauge grid measurement, which is compared to the resistance value printed on the strain gauge data sheet. The measurements were done at the terminal of measurement point, including the jumper wire.
• Resistance between strain gauge and the material the strain gauge is mounted to, insulation resistance.

Strain gauge ID	Strain gauge resistance (Grid A and B)	Insulation resistance
T1 Left arm Upper gauge	A: 350.1 B: 350.4	OL
T1 Left arm Lower gauge	A: 350.5 B: 350.3	OL
T1 Right arm Upper gauge	A: 350.0 B: 349.9	OL
T1 Right arm Lower gauge	A: 350.2 B: 350.3	OL
T2 Left arm Upper gauge	A: 349.8 B: 349.7	OL
T2 Left arm Lower gauge	A: 350.3 B: 350.0	OL
T2 Right arm Upper gauge	A: 350.4 B: 350.1	OL
T2 Right arm Lower gauge	A: 350.9 B: 350.8	OL

Stability check:
All sensors were connected to QuantumX, the signals zeroed, and measurements were made for 10 minutes to check the stability and noise level of the signal. A drift of less than ±10 μm/m is considered acceptable. Test data was recorded and stored for reference. All tests showed acceptable results.
Sample rate: 300 Hz
Automatic Anti-aliasing filter

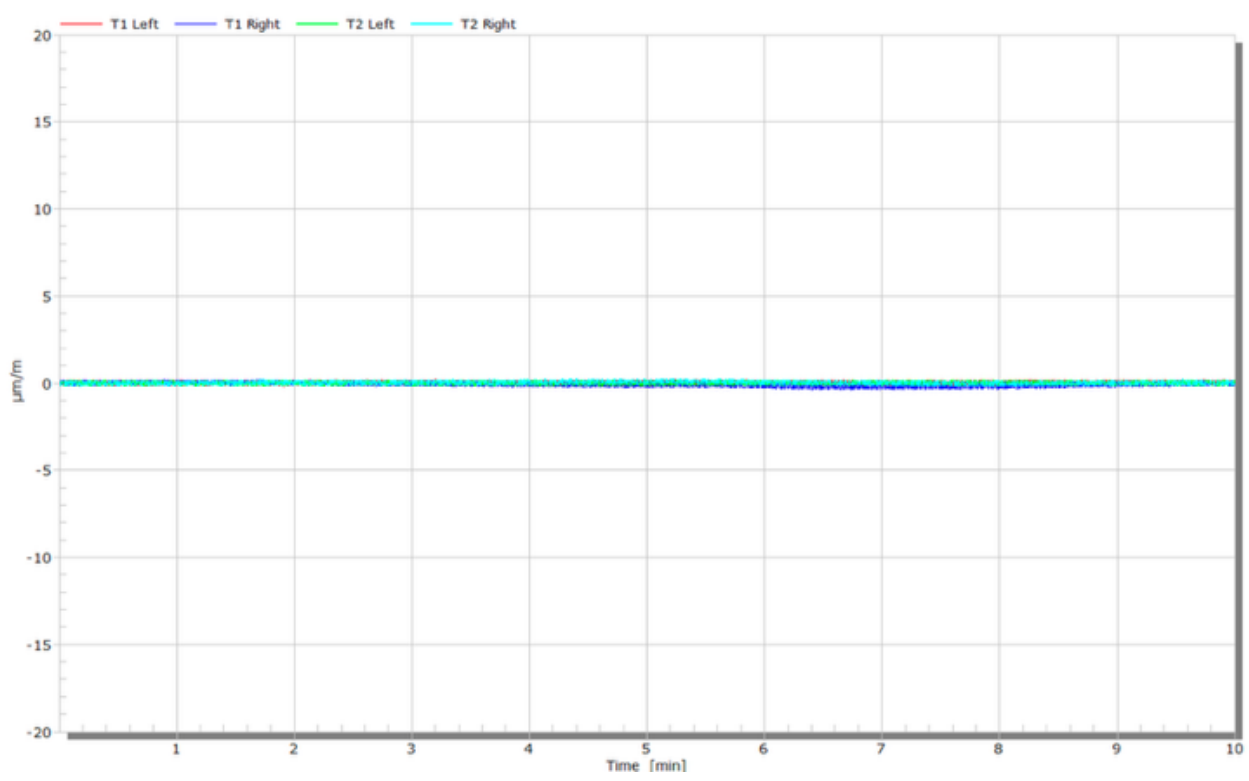

Example output graph showing results of the stability check of the strain gauges

Response check/Calibration:
A load cell was used to correlate strain measurement to a force measurement for each arm. Each arm was loaded with up to 200N of force to create bending in the forceps' arm, as the user would.

The data collected here allowed calibration of each arm resulting in this calibration chart for each individual arm:

Arm ID	Calibration Equation	R^2
T1 Left arm	y = 1.4967x - 0.0984	1
T1 Right arm	y = 1.5124x + 0.7915	0.9995
T2 Left arm	y = 1.4999x - 0.2657	0.9999
T2 Right arm	y = 1.5128x + 0.3984	0.9997

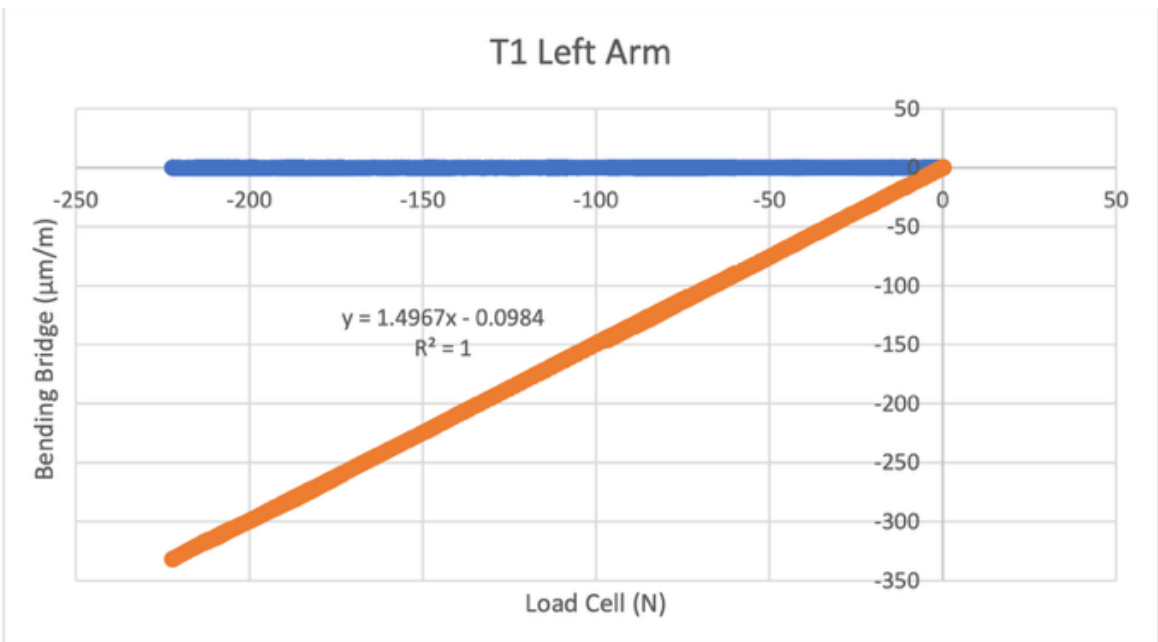

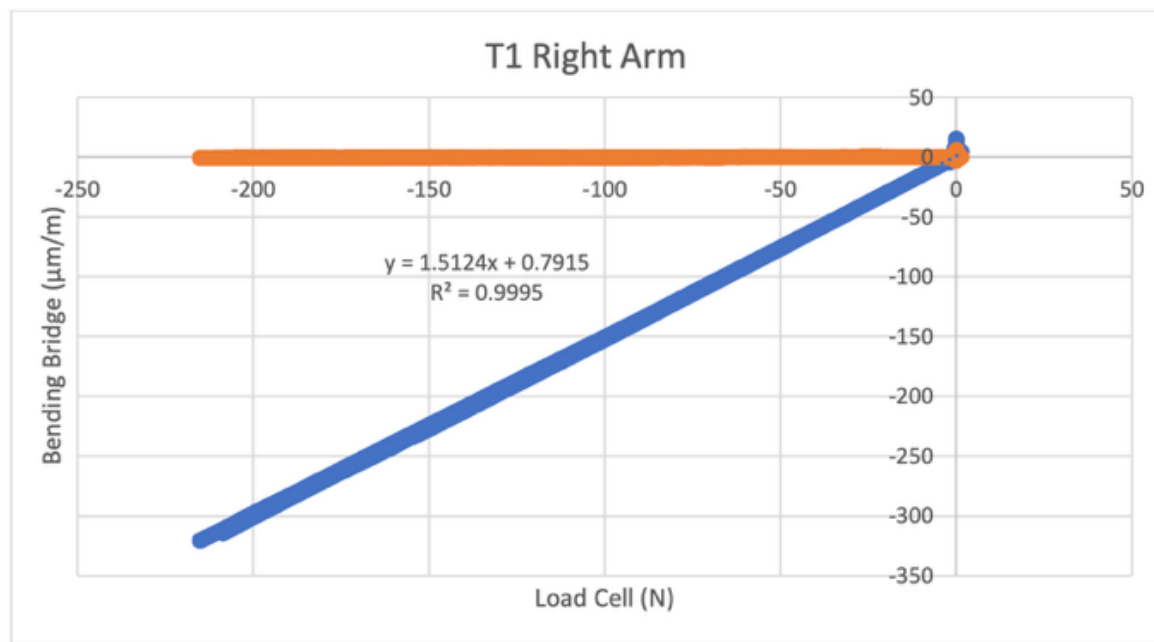

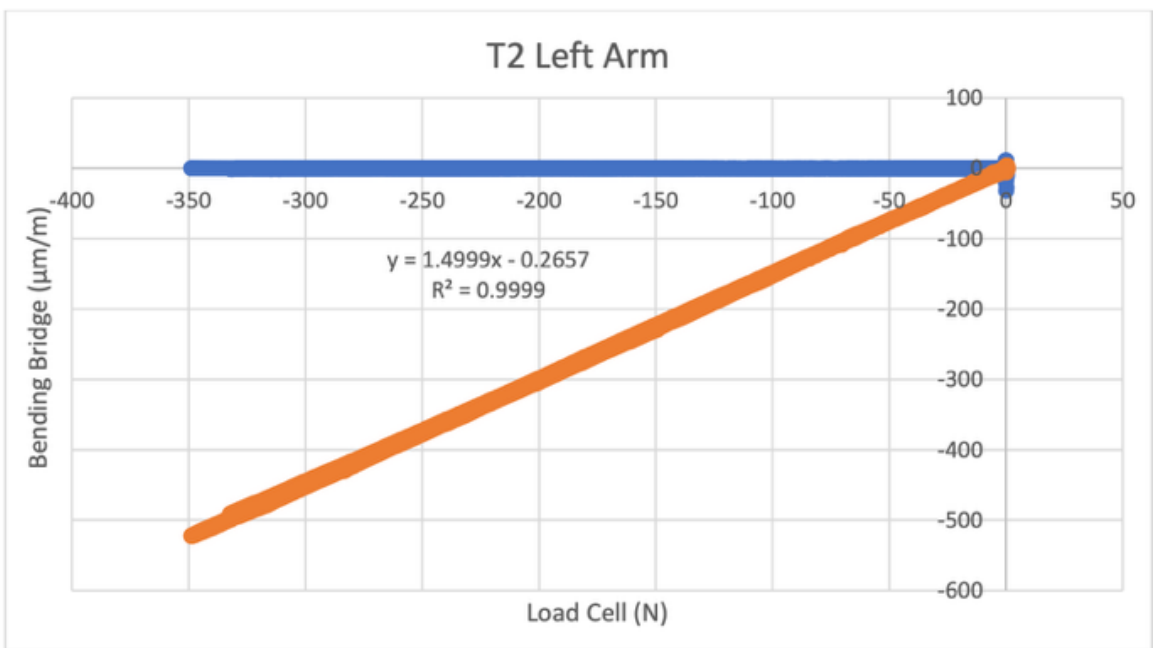

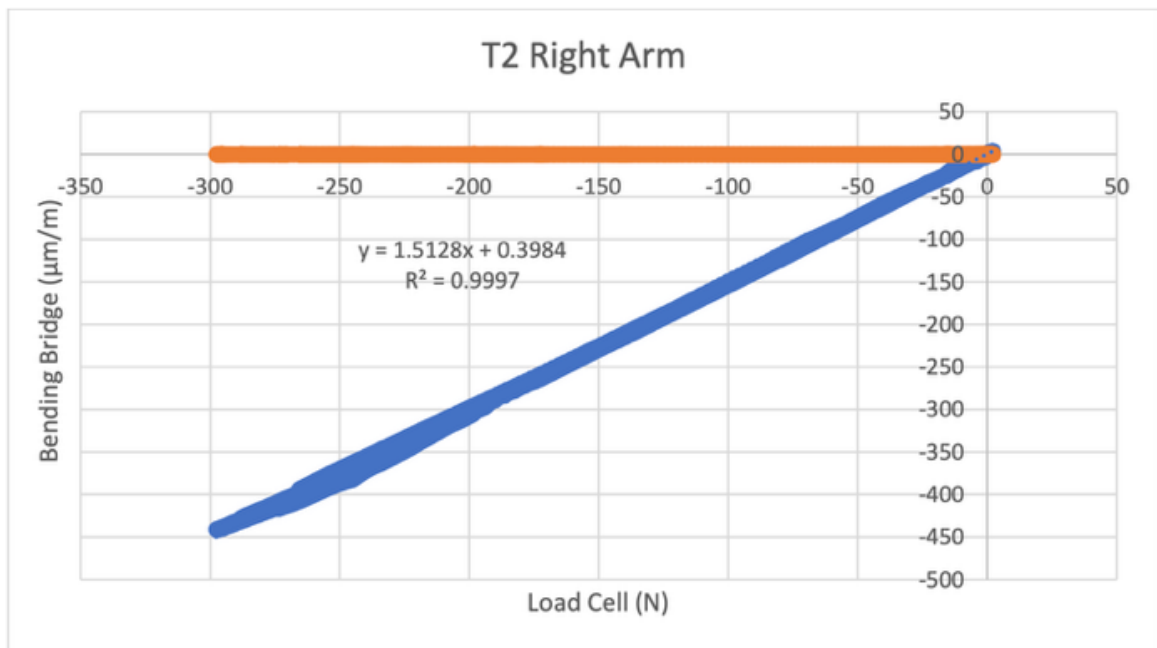

Conclusion
8 strain gauges were installed. They were tested with the QuantumX MX840B amplifier from HBM and measured stable signals. One can estimate the measurement accuracy of the strain gauges to be ±3%.
We can assume then that the sum of the force applied to each arm is the total force necessary to be applied to extract the tooth.

To determine the total force, the following formula is applied:

$$TotalForce\,T1 = \frac{T1left}{1.4967} + \frac{T1right}{1.5124}$$

$$TotalForce\,T2 = \frac{T2left}{1.4999} + \frac{T2right}{1.5128}$$

Test results are provided as graphs. The data is also available upon request.
