# Supplementary material for: A study of the forces generated during equine cheek tooth extraction
Source: Equine Vet J. 2026 Jun 10;58(6):1579–89. doi: 10.1002/evj.70180 (PMC13657190; doi:10.1002/evj.70180)

Equine Veterinary Journal
BEVA

Figure S8: Elevation Times recorded from 22 live cases subdivided by dental arcades. Lower and upper box lines=25th and 75th percentiles, respectively; Middle box line in bold=median; lower and upper whiskers=lower and upper adjacent values, respectively; dots=outliers.

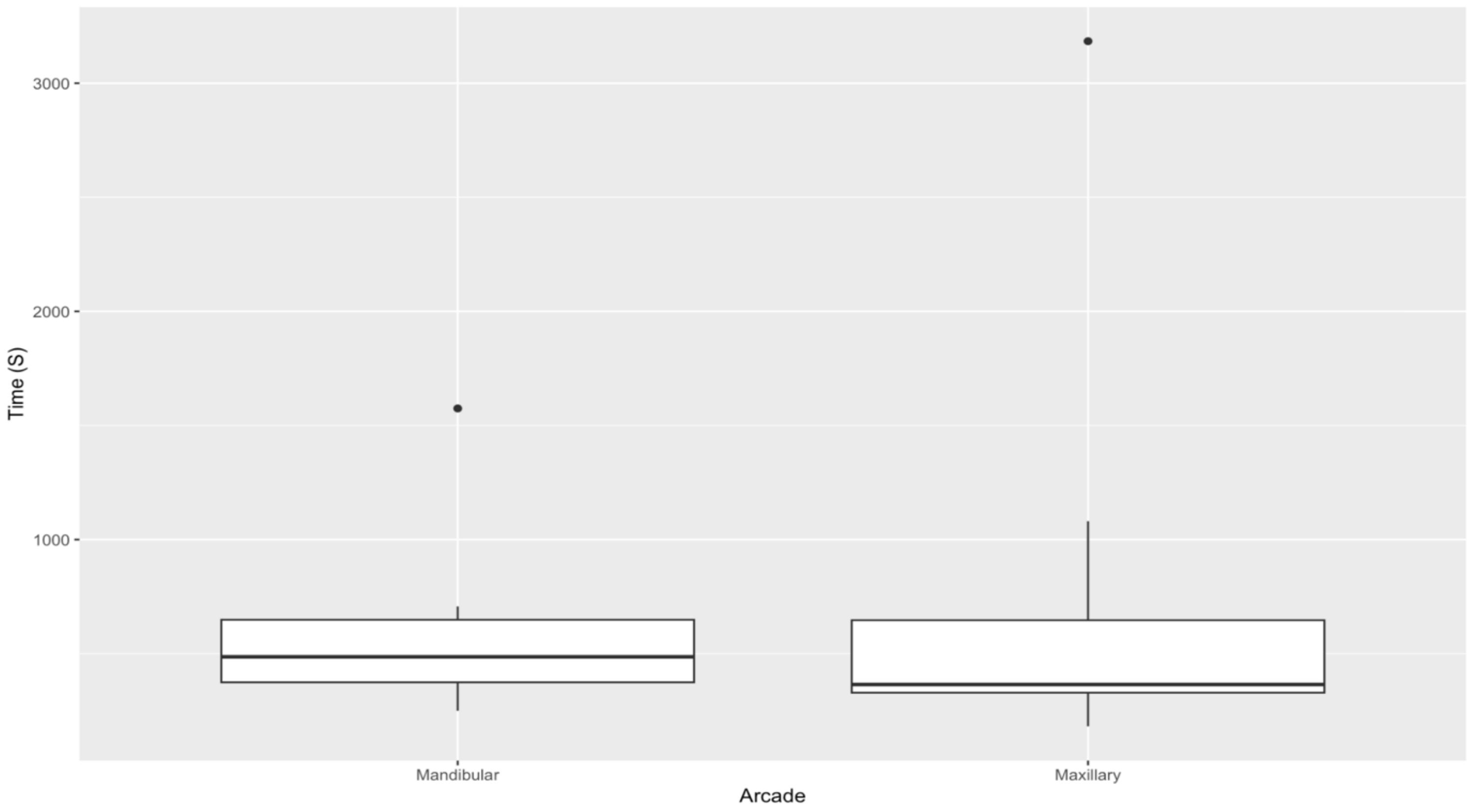

Supplement: Supplementary file 10 [file EVJ-58-1579-s010.pdf]
