# Supplementary material for: A study of the forces generated during equine cheek tooth extraction
Source: Equine Vet J. 2026 Jun 10;58(6):1579–89. doi: 10.1002/evj.70180 (PMC13657190; doi:10.1002/evj.70180)

Equine Veterinary Journal

BEVA

Figure S7: Elevation Impulse recorded from 22 live cases subdivided by age and dental arcades.

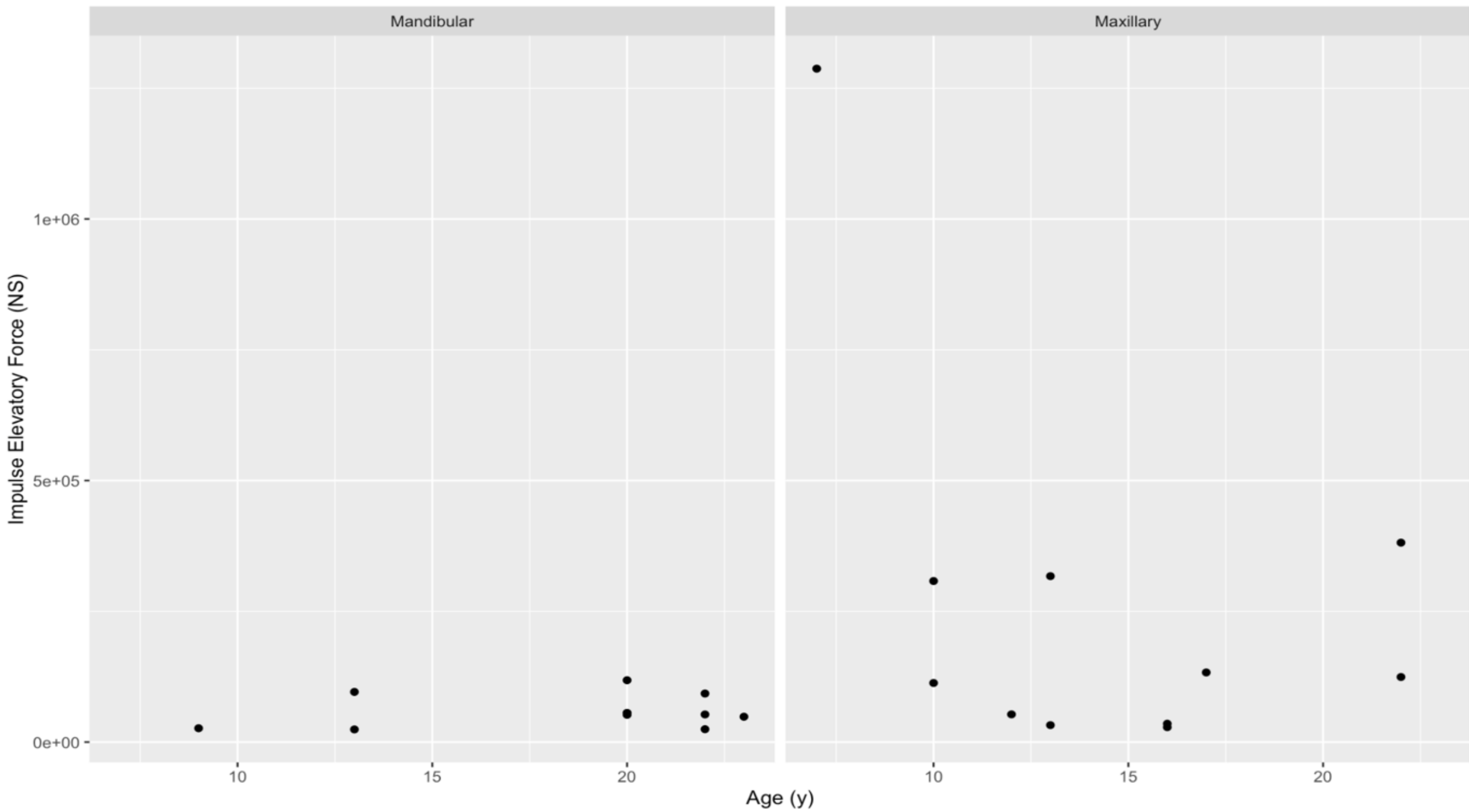

Supplement: Supplementary file 11 [file EVJ-58-1579-s011.pdf]
